# Antibiofilm and Immunomodulatory Effects of Cinnamaldehyde in Corneal Epithelial Infection Models: Ocular Treatments Approach

**DOI:** 10.3390/pharmaceutics18010005

**Published:** 2025-12-19

**Authors:** Ashraf Khalifa, Muthukumar Thangavelu, Krishnaraj Thirugnanasambantham, Hairul-Islam M. Ibrahim

**Affiliations:** 1 Biological Science Department, College of Science, King Faisal University, P.O. Box 400, Al-Ahsa 31982, Saudi Arabia; 2 Department PolymerNano Science and Technology, Jeonbuk National University, 567 Baekje-dearo, Deokjin, Jeonju 54896, Republic of Korea; 3 Faculty of Health and Life Sciences, INTI International University, Nilai 71800, Negeri Sembilan, Malaysia; 4 Department of Microbiology and Immunity, Pondicherry Centre for Biological Sciences and Educational Trust, Pondicherry 605004, India

**Keywords:** ocular drug delivery, cinnamaldehyde, infectious keratitis, biofilm inhibition, host–pathogen interactions, immunomodulation

## Abstract

**Background:** Bacterial keratitis, a major cause of corneal blindness, is frequently associated with biofilm-forming pathogens such as *Klebsiella pneumoniae*. Cyclic-di-GMP (c-di-GMP) controls biofilm development, which increases antibiotic resistance and makes treatment more difficult, highlighting the need for innovative therapeutic approaches. **Methods**: This study investigated cinnamaldehyde as a potential ocular therapeutic using combined computational and experimental approaches. Molecular docking and in vitro assays (XTT, resazurin reduction, crystal violet staining, qRT-PCR, and fluorescence microscopy) were used to evaluate the anti-biofilm and immunomodulatory activities of cinnamaldehyde (CA) against *Klebsiella pneumoniae*. **Results**: CA inhibited biofilm formation in a dose-dependent manner (≈89% at 1000 µM; >50% at 250 µM), reduced bacterial attachment to contact lenses, and downregulated key biofilm genes (*mrkA*, *mrkC*, *ybtS*, *bolA*). Docking analysis revealed strong binding affinity to the *mrkH* regulator (−5.46 kcal/mol. CA maintained more than 80% corneal cell viability by increasing IL-10, suppressing inflammatory mediators (IL-1β, IL-6, and TNF-α), and improving bacterial clearance. **Conclusions**: This study combines computational docking, biofilm quantification, immune cell assays, and functional gene expression analyses to reveal the ability of cinnamaldehyde not only to suppress biofilm formation but also to enhance macrophage-mediated clearance and modulate corneal immune responses, a multi-target approach not previously described in the context of bacterial keratitis. Such effects highlight its potential as a novel ocular drug candidate for protecting corneal integrity in infectious keratitis.

## 1. Introduction

Bacterial keratitis is a microbial infection of the cornea leading to inflammation and potential corneal tissue loss. Although the term “corneal ulcer” is sometimes used interchangeably, bacterial keratitis specifically refers to an infectious etiology, whereas corneal ulcers may also arise from non-infectious causes such as trauma, autoimmune disorders, or neurotrophic keratopathy. With incidence rates ranging from 2.5 to 799 instances per 100,000 people per year, depending on geography and risk factors such contact lens use and ocular trauma, it continues to be a prominent cause of corneal blindness globally [1,2,3]. Prompt diagnosis and targeted therapy are critical to prevent vision loss.

Bacterial keratitis is most frequently associated with risk factors like contact lenses wear, corneal trauma, and preexisting eye surface disorders [2]. In contrast, the etiology of corneal ulcers is more diverse, encompassing both infectious and non-infectious mechanisms [3]. Failure to accurately differentiate between these two conditions may result in misdiagnosis, inappropriate antimicrobial therapy, or delayed identification and treatment of non-bacterial causes. Therefore, comprehensive clinical evaluation—including slit-lamp biomicroscopy and microbiological testing—is essential for accurate diagnosis and effective management [4]. While bacterial keratitis necessitates prompt, targeted antimicrobial therapy, the treatment of non-infectious corneal ulcers centers on addressing the underlying pathology. Recognizing the fundamental differences between bacterial keratitis and corneal ulcers is crucial for ensuring timely, appropriate therapeutic interventions and preventing potential vision-threatening complications [5,6].

*K. pneumoniae* is more widely recognized as a cause of endogenous bacterial endophthalmitis, particularly among Asian populations and those with underlying conditions such as diabetes or liver abscess. There are documented case reports and small case series where *K. pneumoniae* led to severe keratitis or corneoscleral abscess, often with a poor prognosis. Infection leads to complications requiring intensive management and may result in visual loss [7]. *K. pneumoniae* forms biofilms, contributing to infections like corneal ulcer, corneal tissue loss, corneal trauma, ocular surface disease and bloodstream infections [7]. These structured bacterial communities, encased in an extracellular matrix, enhance antibiotic resistance and immune evasion [7].

Increasing resistance among bacterial keratitis pathogens has reduced the efficacy of many first-line antimicrobial agents, necessitating new approaches for treatment. Biofilm formation contributes to chronicity, recurrence, and treatment failure in bacterial keratitis, as it impairs antibiotic penetration and immune clearance.

Microbial adherence to medical devices and tissues, forming biofilms through quorum sensing, leading to persistent infections [8]. Biofilm bacteria are more resistant than free-floating ones, increasing treatment challenges and recurrence risks. Understanding biofilm mechanisms such as adhesins, quorum sensing, and gene regulation is key to developing therapies [9]. Targeting biofilm formation or disruption could improve infection control, but further research is needed [10]. Plant derived secondary metabolites such as phenolics, alkaloids, terpenoids, and flavonoids have shown significant antibacterial and anti-biofilm activity. These metabolites can inhibit biofilm formation, disrupt established biofilms, and act through multiple mechanisms, including altering gene expression, damaging bacterial membranes, inhibiting enzymes, blocking quorum sensing, and affecting cell walls [11]. Their effectiveness depends on factors such as concentration, extraction method, and bacterial strain. Persistent infection and inflammation often result in slow healing, risk of stromal scarring, and vision loss, underscoring the need for agents that can enhance tissue repair while suppressing infection. Few non-antibiotic or adjunct therapies exist that address biofilms and inflammation without cytotoxic effects on the cornea [12].

Cinnamaldehyde (CA), a major bioactive constituent of *Cinnamomum* species, is particularly notable for its safety, chemical stability, and broad-spectrum antimicrobial properties [11]. It is an aromatic aldehyde (C_9_H_8_O) that remains stable under physiological conditions, yet readily forms Schiff bases through reactions with primary amines—an attribute contributing to its diverse biological activities. These physicochemical characteristics underpin its biological efficacy and formulation stability. CA has been widely recognized for its potent antimicrobial and antibiofilm effects against clinically significant bacteria, including *Pseudomonas aeruginosa* and *Staphylococcus aureus*—two major pathogens implicated in bacterial keratitis [13,14]. Several studies have demonstrated cinnamaldehyde’s efficacy at low-to-moderate concentrations, with significant minimum inhibitory concentrations (<500 µM MICs) for Gram-negative bacteria and 62.5 to 250 µ < 250 µM MIC for Gram-positive bacteria [15,16,17]. Moreover, CA has been shown to enhance the activity of conventional antibiotics, such as gentamicin and meropenem, highlighting its synergistic potential against resistant ocular pathogens [18]. Importantly, CA and its delivery systems exhibit low cytotoxicity and good biocompatibility with corneal epithelial cells, supporting its potential for translational application in ocular infections [19]. While immunological reaction when exposed to planktonic *K. pneumoniae* has been extensively studied, our understanding of the immune response to *K. pneumoniae* biofilms remains relatively limited [18].

In *K. pneumoniae*, MrkH is a transcriptional activator that controls the mrk operon’s expression and makes type 3 fimbriae components more easily available. Type 3 fimbriae aid in the colonization of the urinary tract, the attachment of bacteria to human tissues, and the development of biofilms on abiotic surfaces. It is essential to comprehend this operon’s control, especially MrkH’s function, in order to study the pathogenesis of *K. pneumoniae* infections and create plans to prevent biofilm development and combat antibiotic resistance. Because of this, concentrating on MrkH transcriptional activator may be able to halt these procedures and offer substitute methods of preventing *K. pneumoniae* infections [19]. The discovery and development of particular antagonists or inhibitors, however, necessitates a great deal of study, including a full comprehension of the protein’s structure, function, and the molecular mechanisms underlying its activity. A computational method called molecular docking is used in drug discovery to forecast and examine the interactions that may occur when small molecule ligands (potential drugs) attach to a particular target protein. It is essential for the early phases of drug development since it sheds light on the kind of binding, the affinities, and the potential effects of a ligand on the target protein [20].

Hence, cinnamaldehyde emerges as a promising natural compound with broad-spectrum antibiofilm and immunomodulatory activity. Despite its well-documented antibacterial properties against *Pseudomonas aeruginosa*, *Escherichia coli*, and *Staphylococcus aureus* [15,16,17], limited information exists on its efficacy against *Klebsiella pneumoniae* biofilms in ocular contexts or its influence on host immune responses during biofilm-associated infection. The *mrk* operon of *K. pneumoniae*, regulated by the transcriptional activator MrkH, plays a central role in type 3 fimbriae production, surface adhesion, and biofilm establishment [20]. Targeting MrkH and related virulence determinants could disrupt biofilm development and enhance host clearance mechanisms.

In this study, the novelty lies in demonstrating, for the first time, the dual action of cinnamaldehyde as both a biofilm inhibitor and an immunomodulatory agent against biofilm-forming *K. pneumoniae* in ocular infection models. We specifically investigated its antibiofilm efficacy and its ability to modulate key biofilm-related genes (*mrkH*, *mrkA*, *ybtS*), alongside assessing its effects on host immune responses, including macrophage-mediated bacterial clearance and corneal cell migration. Using a combination of computational docking, in vitro biofilm inhibition assays, and macrophage functional analyses with the RAW 264.7 model, this study integrates antimicrobial and host-directed strategies to uncover new therapeutic insights.

We hypothesize that cinnamaldehyde exerts a dual biological action by (i) inhibiting *K. pneumoniae* biofilm formation through modulation of biofilm-related gene expression, and (ii) enhancing host immune responses, thereby improving bacterial clearance and corneal tissue repair. This integrative approach provides novel mechanistic understanding of cinnamaldehyde as a natural, non-antibiotic therapeutic candidate for managing biofilm-associated bacterial keratitis.

## 2. Materials and Methods

### 2.1. Chemicals, Microbial Strains and Clture Conditions

The bacterial strains used in this work included *Pseudomonas aeruginosa* (ATCC 27853), methicillin-resistant *Staphylococcus aureus* (MRSA, ATCC 43300), and *Klebsiella pneumoniae* (MTCC 109), obtained from the American Type Culture Collection and the Institute of Microbial Technology (Chandigarh, India). Stock cultures were maintained at 4 °C on Nutrient Agar (NA; Himedia, Mumbai, India; cat. no. MM012). For experimental use, a single *K. pneumoniae* colony was inoculated into brain–heart infusion (BHI) broth (Himedia, Mumbai, India; cat. no. M210) supplemented with 2% glucose and incubated overnight at 37 °C with shaking at 200 rpm to achieve an actively growing culture. Trans-cinnamaldehyde (Tca; 99% purity; cat. no. C80687) was obtained from Sigma, Delhi, India, and all other media and reagents, unless stated otherwise, were sourced from Himedia (Mumbai, India).

### 2.2. Antibacterial Activity of Trans-Cinnamaldehyde Against K. pneumoniae

The antibacterial activity of cinnamaldehyde against *P. aeruginosa*, MRSA, and *K. pneumoniae* was investigated using a disk-diffusion method [21]. *K. pneumoniae* was grown at 37 °C for 24 h on Nutrient Agar (Himedia, Mumbai, India). Bacterial inoculations equivalent to 0.5 McFarland (1 × 10^8^ CFU/mL) were made in antiseptic saline (0.85% NaCl) (*w*/*v*) solution. On Mueller-Hinton agar (MHA) plates, the aforementioned suspension was dispersed. A sterile disk of diameter of 6 mm was placed, and trans-cinnamaldehyde (250, 500, and 1000 µM) was dissolved in 5% DMSO was added into the well. After adding the suggested standard antibacterial agent ciprofloxacin (20 µg/disk) to each well, the plates were incubated for 24 h at 37 °C. By measuring the zone of inhibition (mm in diameter), the activity against *K. pneumoniae* was ascertained.

### 2.3. Minimal Inhibitory Concentration (MIC)

Using micro-dilution of broth on microtitre plates, the least inhibitory concentrations (MICs) of planktonic suspended cells for *P. aeruginosa*, MRSA and *K. pneumoniae* were identified [22]. Aseptic techniques were used to prepare the plates. Each well received 100 µL of ciprofloxacin (20 µg/mL) and cinnamonaldehyde (1 mM) in 1% (*v*/*v*) DMSO (DMSO-treated biofilms as the experimental reference) or sterile water, which were then serially diluted to lowest possible concentrations of (62.5 µM) and (1.25 µg/mL), respectively, of the two chemicals. The wells were then filled with 10 µL of bacterial suspension (1 × 10^6^ CFU/mL), which was then incubated for 24 h at 37 °C. Following incubation, each well received 30 µL of resarzurin (0.015%), which was then incubated for a further 2–4 h to see any color changes. The concentration of trans-cinnamaldehyde/ciprofloxacin that inhibited cell growth was then designated as MIC after comparing the well color.

### 2.4. Biofilm Eradication Assay Using CV and XTT

The metabolic and biomass inhibitory capacity of CA on *P. aeruginosa*, MRSA, and *K. pneumoniae* biofilm was assessed using crystal violet staining and the XTT assay was carried out using *P. aeruginosa*, MRSA, and *K. pneumoniae*. Each strain was grown overnight, and the resulting cultures were adjusted to approximately 1 × 10^6^ CFU/mL in BHI broth supplemented with 2% (*w*/*v*) glucose. Two hundred microliters of the standardized bacterial suspension were dispensed into each well of a 96-well flat-bottom microplate, followed by the addition of cinnamaldehyde at concentrations ranging from 62.5 to 1000 µM. The plates were incubated at 37 °C for 72 h. Media-only and untreated culture controls were included in all experiments.

For the XTT assay, 100 µL of fresh sterile medium was added to each well. A 50 µL aliquot of the XTT reaction mixture, prepared with phenazine methosulfate (PMS) and XTT (2,3-Bis-(2-Methoxy-4-Nitro-5-Sulfophenyl)-2H-Tetrazolium-5-Carboxanilide), was then dispensed into every well. The plates were incubated at 37 °C for 4 h to allow the colorimetric reaction to develop. After incubation, the microplates were gently agitated for approximately 10 s to ensure uniform mixing of the dye, and absorbance was subsequently recorded at 450 nm. To assess the impact of cinnamaldehyde on *K. pneumoniae* biofilm formation, 100 µL of 0.1% (*w*/*v*) crystal violet solution was added to each well for the staining step. The empty wells incubated for 10 min. Following the removal of the free crystal violet solution, sterile distilled water was used to wash each well. Then, each well was filled with 95% ethanol, and the plate was shaken at room temperature for 30 min to measure the absorbance of color intensity at 595 nm [23]. The amount of biofilm inhibition was measured, using Biotek microplate reader (Biotek, Calabasas, CA, USA) and the minimal biofilm inhibitory concentration (MBIC) was set at 80% suppression of biofilm development [24].

### 2.5. Bacterial Biofilm Attachment and Proliferation in Contect Lens

#### 2.5.1. Biofilm Evaluation Using Fluorescence Studies

Fluorescence imaging was performed to evaluate the impact of cinnamaldehyde on the biofilms formed by *P. aeruginosa*, MRSA, and *K. pneumoniae*, following previously reported procedures with minor modifications [25]. Biofilms were established in 12-well culture plates and exposed to CA at concentrations ranging from 62.5 to 1000 µM in BHI broth supplemented with 2% (*w*/*v*) glucose. Cultures were incubated at 37 °C for 72 h. After incubation, each well was carefully rinsed with sterile phosphate-buffered saline (PBS) to remove planktonic cells. The biofilms were then stained by adding acridine orange (5 µL, 10 mg/dL; CAS 10127-02-3, Biosynth Ltd., Berkshire, UK) and allowing the dye to interact with the cells for 30 min. Fluorescent images were captured using an Optika fluorescence microscope (Optika, Ponteranica, Italy). For each sample, five randomly selected fields were documented, and all imaging parameters—including illumination intensity, background settings, and contrast—were maintained consistently across experiments.

#### 2.5.2. Quantification of Nitric Oxide in the HCEC–Biofilm Co-Culture System

Human corneal epithelial cells (HCEC; 1 × 10^5^ cells/well) were seeded in 12-well plates and allowed to adhere for 6 h at 37 °C in a humidified atmosphere containing 5% CO_2_, using serum- and antibiotic-free DMEM. The wells were then exposed to preformed biofilms of *P. aeruginosa*, MRSA, and *K. pneumoniae* as previously described. Following cell attachment, the co-culture system was treated with cinnamaldehyde (250 µM), selected based on its >50% biofilm inhibitory activity. After 24 h of incubation, culture supernatants were collected and transferred into a fresh 96-well microplate for nitric oxide (NO) analysis. Equal volumes of the Griess reagent (1% sulfanilamide and 0.1% naphthyl-ethylenediamine dihydrochloride prepared in 2.5% phosphoric acid) were mixed with the samples and incubated for 10 min at room temperature. Nitrite levels were quantified by measuring absorbance at 540 nm using a Biotek microplate reader (Biotek, CA, USA).

### 2.6. Quantification of Internalized Bacteria (Antibiotic Protection Assay)

An antibiotic protection assay was employed to quantify the number of bacterial cells internalized by macrophages [26]. RAW 264.7 cells were detached using a sterile cell scraper (Himedia, India), subcultured, and maintained in DMEM supplemented with 10% fetal bovine serum (FBS) (Himedia, Mumbai, India). Macrophages (1 × 10^5^ cells/well) were then co-incubated with pre-established biofilms of *P. aeruginosa*, MRSA, or *K. pneumoniae* (1 × 10^6^ CFU/mL; grown for 72 h at 37 °C in BHI medium containing 2% glucose), either alone or in the presence of non-cytotoxic concentrations of trans-cinnamaldehyde, following the procedure described by [16]. The co-culture was set up in 12-well plates and incubated for 90 min at 37 °C in a 5% CO_2_ atmosphere using serum- and antibiotic-free DMEM. After the 90 min uptake period, the medium was replaced with DMEM containing 10% FBS and an antibiotic–antimycotic mixture (Himedia, Mumbai, India) to eradicate extracellular bacteria, and the cells were further incubated for 12 h. The macrophages were then washed three times with sterile PBS and lysed with 0.1% Triton X-100 for 5 min to release intracellular bacteria. Serial dilutions of the lysates were plated onto Brain Heart Infusion agar (Himedia, Mumbai, India) and incubated aerobically for 24 h at 37 °C. Colony counts were used to determine the number of viable intracellular bacteria, expressed as CFU per 10^5^ macrophages.

### 2.7. HCEC Corneal Cell Migration Assay

Human corneal epithelial cells (HCEC) were obtained from the National Centre for Cell Science (NCCS), Pune, India, and maintained in DMEM supplemented with 10% FBS at 37 °C in a 5% CO_2_ incubator. The migratory behavior of HCEC was evaluated using a standard Transwell assay (24-well format, 8 µm pore inserts; Himedia, Mumbai, India) as previously described [27]. To generate biofilms for the assay, *P. aeruginosa*, *S. aureus*, and *K. pneumoniae* cultures (1 × 10^5^ CFU/mL) were added to the lower chambers containing BHI broth enriched with 2% (*w*/*v*) glucose. Plates were kept under static conditions at 37 °C for 72 h to allow biofilm establishment. After incubation, the medium and non-adherent bacteria were removed, and each well was thoroughly washed with sterile PBS. Fresh serum-free, antibiotic- and antimycotic-free DMEM containing 250 µM cinnamaldehyde was then added to the lower chamber. Macrophages (5 × 10^5^ cells) were seeded into the upper inserts. For negative controls, only DMEM (without biofilm or cinnamaldehyde) was added to the lower wells. The Transwell plates were incubated for 12 h at 37 °C in 5% CO_2_ to permit cell migration. At the end of the incubation, non-migrated cells on the upper surface of the insert membrane were removed gently using a sterile swab, followed by a light rinse with PBS. The underside of the membrane was fixed in 4% paraformaldehyde for 30 min and subsequently stained with 0.1% crystal violet for 15 min in the dark. Membranes were rinsed with distilled water, and migrated cells were imaged using a phase-contrast microscope (Optika, Ponteranica, Italy).

The migration index (%) was calculated using the following formula:

Migration Index (%) = [stained cell count (s)/stained cell count (−)] × 100%(1)

where stained cell count (−) represents macrophage numbers after co-culture with DMEM alone and stained cell count (s) represents macrophage numbers after co-culture with bacterial biofilms or with cinnamaldehyde-treated biofilms.

### 2.8. RNA Extraction, cDNA Synthesis, and Quantitative Real-Time Polymerase Chain Reaction (qRT-PCR)

For the gene expression study, *K. pneumoniae* biofilms were prepared by inoculating cultures at 1 × 10^6^ CFU/mL into 12-well plates containing BHI broth supplemented with 2% (*w*/*v*) glucose. The plates were incubated at 37 °C for 72 h under static conditions to allow biofilm establishment. After biofilm formation, the wells were carefully replenished with 2 mL of either trans-cinnamaldehyde (250 µM) in BHI (treatment group) or BHI containing an equivalent volume of DMSO (control group). Cultures were then incubated for an additional 24 h at 37 °C. Total RNA was isolated from both untreated and cinnamaldehyde-exposed biofilms using RNAiso Plus reagent (Takara Bio Inc., Osaka, Japan) according to the manufacturer’s protocol. First-strand cDNA was synthesized from the extracted RNA using the PrimeScript™ 1st Strand cDNA Synthesis Kit (Takara Bio Inc., Osaka, Japan). The reaction mixture was brought to a final volume of 20 µL with RNase-free water after adding 1 µL of PrimeScript reverse transcriptase (200 U/µL). Reverse transcription was performed following the recommended temperature profile: 30 °C for 10 min, 42 °C for 60 min, and 95 °C for 5 min to terminate the reaction. To assess the transcriptional response of macrophage immune-related genes, RAW 264.7 macrophages were co-cultured with *K. pneumoniae* biofilms as described in the Antibiotic Protection Assay. After 6 h of co-incubation, the cells were washed with DEPC-treated PBS, and total RNA was extracted using TRIzol reagent. cDNA synthesis was carried out using the PrimeScript™ RT-PCR Kit (Takara, Osaka, Japan), following the manufacturer’s guidelines. Quantitative real-time PCR was performed using 2× SYBR^®^ Select Master Mix (Applied Biosystems, Foster City, CA, USA). The primer sets used for bacterial and macrophage genes are listed in Table 1 and Table 2.

Amplification reactions were run on a Rotor-Gene Q 2PLEX HRM Real-Time PCR system (Qiagen, Venlo, The Netherlands). Relative mRNA expression was calculated using the 2^−ΔΔCT^ method [27], with 16S rRNA serving as the bacterial reference gene and GAPDH as the housekeeping gene for RAW 264.7 macrophages. Each cDNA sample was analyzed in triplicate across three independent experiments.

### 2.9. Computational Analysis of MrkH and Cinnamaldehyde Interactions

Molecular docking studies were performed using AutoDock v4.2 in combination with AutoDockTools (ADT) v1.5.4 [28,29]. The ligand, cinnamaldehyde (CID_637511), was retrieved from the PubChem database. Three-dimensional crystal structures of the target proteins—including the *P. aeruginosa* and MRSA proteins, as well as *K. pneumoniae* MrkH, a c-di-GMP–responsive transcriptional regulator—were downloaded from the RCSB Protein Data Bank (PDB ID: 5KGO). Docking was conducted by treating the receptor as a rigid molecule, while allowing full torsional flexibility for the ligand. Entire receptor structures were used for blind docking analyses. The search for optimal binding conformations employed the Lamarckian genetic algorithm, using a population size of 150, a mutation rate of 0.02, and five generations of refinement. Docking outcomes were ranked based on predicted binding energy values. Cluster analyses were subsequently performed using RMSD measurements relative to the native protein geometry. The conformation with the lowest binding energy within the most populated cluster was considered the most reliable docking solution. Ligand–receptor interaction profiles were visualized and interpreted using the Discovery Studio 2021 Client (trial version).

### 2.10. Statistical Analysis

All experiments were performed in technical triplicates and repeated across a minimum of three independent biological replicates to ensure analytical robustness. Quantitative outcomes are reported as mean ± SEM. Statistical analyses were conducted using GraphPad Prism 5. Assumption testing included the Shapiro–Wilk test for distributional normality and Levene’s test for homogeneity of variances. For two-group comparisons, an unpaired, two-tailed Student’s *t*-test was applied, with Welch’s correction used when variance equality was violated. Multi-group comparisons were analyzed using one-way ANOVA followed by Tukey’s HSD post hoc test to control the family-wise type I error rate. Effect size metrics (Cohen’s d for pairwise contrasts and η^2^ for ANOVA models) were calculated to quantify the magnitude of treatment effects. Ninety-five percent confidence intervals were reported to indicate the precision of point estimates. Statistical significance was defined a priori as *p* < 0.05. Exact *p*-values, test statistics (t, F), and degrees of freedom are provided in tables or figure legends to support transparency and reproducibility.

## 3. Results

### 3.1. Trans-Cinnamaldehyde Antibacterial Properties Against S. aureus, K. pneumoniae, and P. aeruginosa

**Figure 1:**
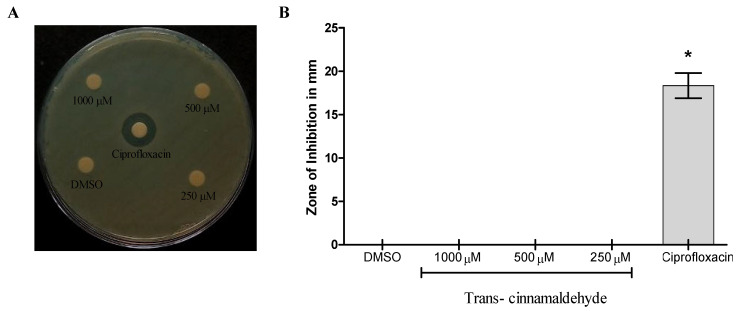
Cinnamaldehyde’s antimicrobial activity against *K. pneumonia* was studied using the agar well disk diffusion method. (**A**) The zone of inhibition (mm) of samples was compared to the standard Ciprofloxacin (20 µg/well) against *K. pneumonia*. (**B**). Statistical significant differences as compared between CA 250 µM to 1000 µM with DMSO and positive control (Ciprofloxacin) groups. Values were reported as means ± standard error for all three replicates, and * *p* ≤ 0.05 indicated significant results.

According to the current investigation, cinnamaldehyde did not show any antibacterial activity against *K. pneumoniae* in any of the investigated doses, even at the maximum concentration of 1000 µM (Figure 1A,B). Furthermore, cinnamaldehyde had no antibacterial efficacy against *P. aeruginosa*, *S. aureus*, and *K. pneumonia*, according to a minimum inhibitory concentration (MIC) investigation using resazurin at its highest test concentration (1000 µM) (Figure S1).

### 3.2. Biomass and Metabolic Inhibitory Potential of CA Against K. pneumoniae Biofilm

**Figure 2:**
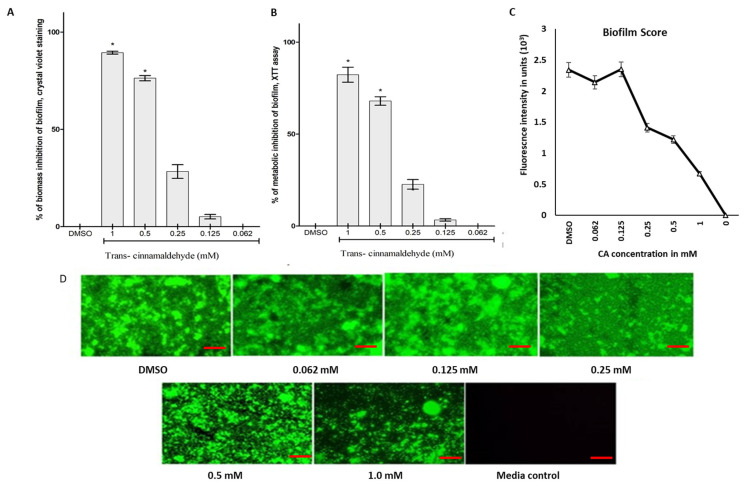
The impact of trans-cinnamaldehyde on the biofilm of *K. pneumoniae*. The *K. pneumoniae* biofilm were quantitatively measured using three different methods: (**A**). crystal violet staining; (**B**). XTT reduction assay; and (**C**,**D**). Acridine orange fluorescence quantification unit and staining for biofilm assessment. The vehicle control utilized was DMSO. The red line scale bar represents 100 µM. Statistical significant differences as compared between CA with DMSO groups and the findings were shown as the means ± standard error for the three repetitions. * *p* ≤ 0.05 was used to assess the data’ significance.

According to the findings, cinnamaldehyde exhibited a dose-dependent suppression of K. pneumoniae biofilm formation. The crystal violet staining revealed that CA was able to prevent the *K. pneumoniae* biofilms starting at a concentration of 125 µM. At a concentration of 1000 µM, CA was reported to inhibit the production of *K. pneumoniae* biofilms by 89% (Figure 2A). The XTT reduction assay demonstrated a similar pattern of biofilm inhibition by cinnamaldehyde (Figure 2B). These data indicate that CA can suppress the development of *K. pneumoniae* biofilms in a manner that is dose-dependent. Both the CV and XTT reduction assay provide supporting evidence for the biofilm inhibitory effects of cinnamaldehyde. Furthermore, as previously reported, CA 250 µM inhibited the formation of ocular biofilms more than 50% and it was confirmed by the fluorescent staining using acridine orange (Figure 2C).

### 3.3. CA Inhibits the Cell Attachment and Bacterial Load of Ocular Microbes in HCEC Cell Lines

**Figure 3:**
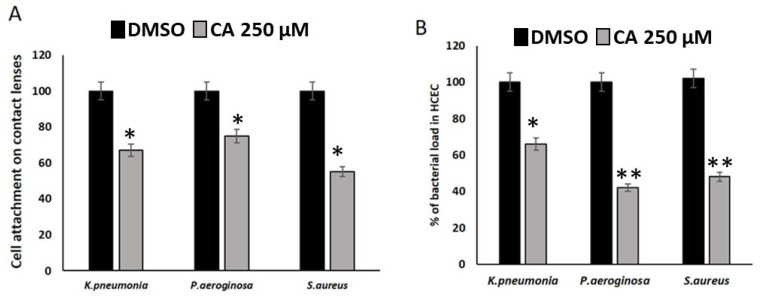
Cell attachment and bacterial load in HCEC cell lines. (**A**) Cell attachment of three bacterial species—*K. pneumoniae*, *P. aeruginosa*, and *S. aureus*—to contact lenses following treatment with either DMSO (black bars) or Cinnamaldehyde (CA) 250 µM (gray bars). Cell attachment is expressed as a percentage relative to the DMSO control (mean ± SD). (**B**) Percentage of bacterial load in human corneal epithelial cells (HCEC) after exposure to contact lenses treated with DMSO (black bars) or Cinnamaldehyde (CA) 250 µM (gray bars). Bacterial load is shown as a percentage relative to the DMSO control (mean ± SD). CA 250 µM significantly reduced both bacterial attachment to contact lenses and subsequent bacterial load in HCEC for all tested species compared to DMSO. Statistical significant differences as compared between CA with DMSO groups and the results were reported as the means ± standard error of three replicates. * *p* ≤ 0.05 and ** *p* ≤ 0.01 were used to determine significance in the results.

The results presented in Figure 3 demonstrate that treatment with CA 250 µM significantly reduces both bacterial attachment to contact lenses and subsequent bacterial load in human corneal epithelial cells (HCEC) compared to the DMSO control across all tested bacterial species. Specifically, Figure 3A shows that the percentage of cell attachment on contact lenses by *K. pneumoniae*, *P. aeruginosa* and *S. aureus* is markedly lower in the presence of CA 250 µM concentration (gray bars) than with DMSO (black bars). For example, *S. aureus* attachment is reduced from approximately 100% in the DMSO group to about 55% with CA 250 µM concentration. Similar trends are observed for *K. pneumoniae* and *P. aeruginosa*, with reductions to approximately 65% and 75%, respectively. Figure 3B further reveals that the bacterial load in HCEC after exposure to treated contact lenses also decreases significantly with CA 250 µM concentration. The reduction is most pronounced for *P. aeruginosa*, where bacterial load drops to about 45% compared to the DMSO control. *K. pneumoniae* and *S. aureus* show reductions to approximately 65% and 50%, respectively. These findings indicate that compound 500 is effective in inhibiting both the initial bacterial colonization of contact lenses and the subsequent infection risk to corneal epithelial cells, suggesting its potential utility in preventing contact lens-associated eye infections.

### 3.4. Modulation of HCEC Epithelial Cell Lines Migration Response by Cinnamaldehyde

**Figure 4:**
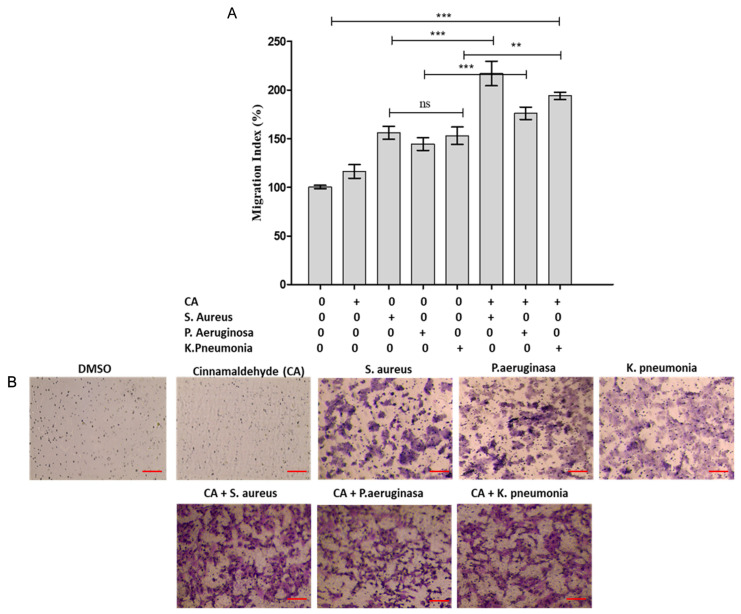
Effect of cinnamaldehyde on migration of HCEC epithelial co-cultured with ocular biofilm. (**A**) HCEC epithelial cell line migratory index; (**B**) Bright-field images of migrated HCEC cell lines co-cultured with biofilm of *P. aeruginosa*, *S. aureus* and *K. pneumoniae* treated with or without cinnamaldehyde. Statistically significant differences as compared between cinnamaldehyde with DMSO groups. The above data represents the mean ± standard error of triplicate values. The red line scale bar represents 50 µM. The results were considered significant for ns (Not significant), ** *p* ≤ 0.01; *** *p* ≤ 0.001.

Corneal epithelial cell lines, which are stimulated to migrate by cinnamaldehyde, serve as the first line of defense against *P. aeruginosa*, *S. aureus* and *K. pneumoniae* and have the capacity to eradicate the bacteria. After being exposed to cinnamaldehyde, macrophages respond rapidly. The migration of HCEC alone or co-cultured with *K. pneumoniae* near the bottom of the membrane after treatment with cinnamaldehyde or DMSO was detected by crystal violet staining, as shown in Figure 4. The number of migrating cells in the wells treated with bacteria-free cinnamaldehyde and the DMSO group does not significantly differ from one another. However, it was discovered that co-culturing with *K. pneumoniae* biofilm significantly increased macrophage movement. CA 250 µM treatment dramatically increased the macrophage migration that were co-cultured with *P. aeruginosa*, *S. aureus* and *K. pneumoniae* biofilm (Figure 4).

### 3.5. CA Inhibited the Expression of Genes Involved in the Biofilm Formation of K. pneumoniae

**Figure 5:**
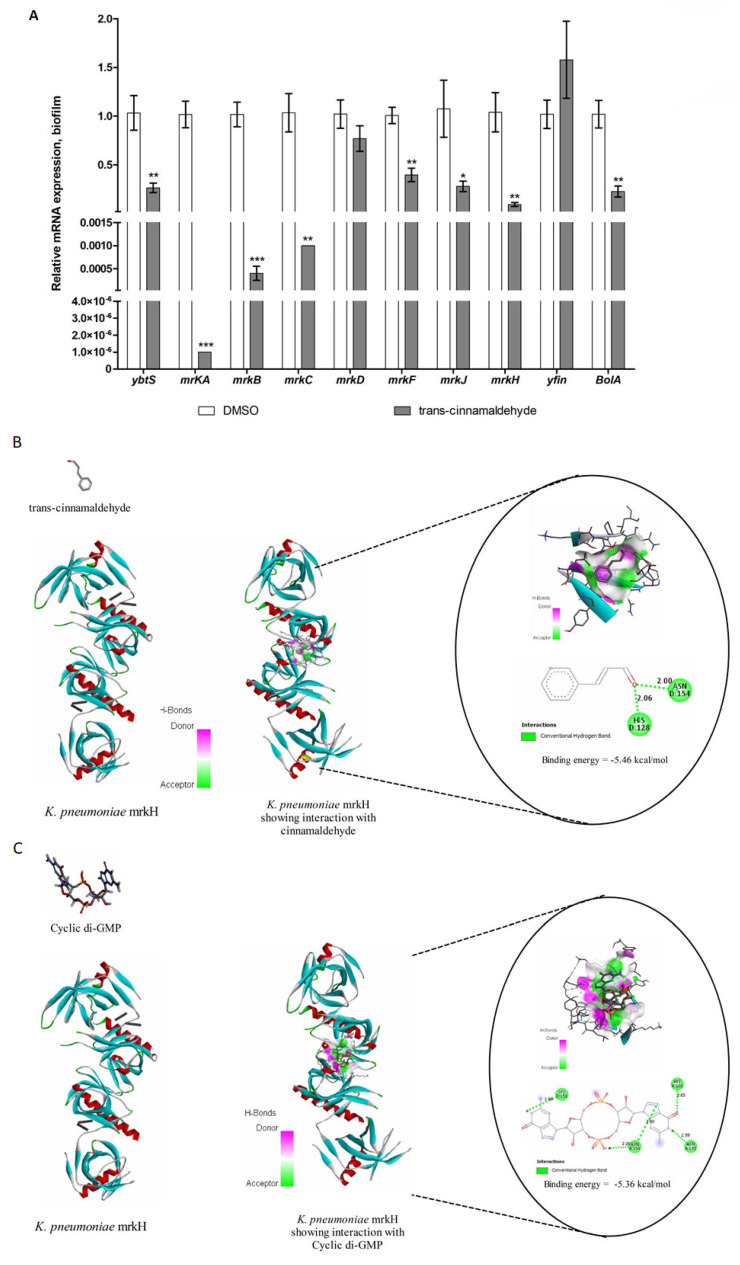
Regulation and interaction of CA on biofilm markers. (**A**) Quantitative real time PCR analysis of genes involved in *K. pneumoniae* biofilm formation. Statistical significant differences as compared between cinnamaldehyde with DMSO groups and results were expressed as mean relative expression ± standard error of three replicates. (**B**,**C**). Interaction of cinnamaldehyde to the active site of *K. pneumoniae* c-Di-GMP-Regulated transcription factor (mrkH). The results were considered significant for * *p* ≤ 0.05; ** *p* ≤ 0.01; *** *p* ≤ 0.001.

In an effort to comprehend the molecular underpinnings of cinnamaldehyde-mediated suppression of *K. pneumoniae* biofilm development, we employed qRT-PCR to investigate the expression patterns of genes associated with biofilm formation and virulence (Figure 5). According to the findings of the qRT-PCR investigation, the expression of *ybtS*, which codes for salicylate synthase involved in the production of yersiniabactin by *K. pneumoniae*, was considerably downregulated upon treatment with cinnamaldehyde (*p* < 0.01) (Figure 5). CA treatment also downregulated the expression of mrkA, which encodes the main subunit of the Type 3 fimbrial shaft, in *K. pneumoniae* biofilms (*p* < 0.001). Ocular Biofilms of *K. pneumoniae* treated with CA also showed downregulated expressions of mrkB mRNA, which codes for chaperone (*p* < 0.001), and mrkC mRNA, which codes for outer membrane usher (*p* ≤ 0.01). However, in *P. aeruginosa*, *S. aureus* and *K. pneumoniae* biofilms, cinnamaldehyde had no effect on the expression of mrkD mRNA, which codes for fimbrial adhesion protein (*p* > 0.05), and yfin mRNA, which encodes diguanylate cyclase (*p* > 0.05). Expression of mrkF (*p* < 0.01), encoding a protein involved in assembly of the filament, and mrkJ (*p* ≤ 0.05), encoding phosphodiesterase, in the biofilms of *K. pneumoniae* were also downregulated by cinnamaldehyde treatment. In addition, cinnamaldehyde treatment downregulated the expression of mRNA-encoding BolA protein (*p* < 0.01) in the biofilms of *K. pneumonia*.

### 3.6. Computational Docking and Interaction of mrkH with CA and C-Di-GMP

The purpose of this investigation was to examine the relationship between cinnamaldehyde and *K. pneumoniae* mrkH. Using molecular docking tools, we present the binding ability of cinnamon aldehyde to *K. pneumoniae* mrkH. All of the hydrogen bonds, hydrogen bond lengths, and molecular docking binding energies (kcal/mol) associated with the c-Di-GMP interaction (Figure 5C) and cinnamaldehyde:mrkH (Figure 5B) have been recorded. The biofilm inhibiting activity of cinnamaldehyde may be explained by the binding energy values of −5.46 (kcal/mol) for cinnamaldehyde and −5.36 (kcal/mol) for c-Di-GMP against the mrkH protein in the aforementioned interaction. Figure 5B,C depict the various hydrogen bond interaction, hydrogen bond receptor-side surface interaction, and 2D-hydrogen bond interface representations. Four hydrogen bonds between c-Di-GMP and HIS128, ASN133, LYS151, and ASN154 were discovered through docking experiments (Figure 5C). Likewise, HIS128 and ASN154 in mrkH participate in ligand-protein interaction with cinnamaldehyde (Figure 5B). The aforementioned findings demonstrated that cinnamaldehyde and c-Di-GMP had the same binding site in mrkH. Binding of CA to the biofilm bacteria quorum-sensing targets Rhl1 and Las1 showed that CA strongly attracts these targets and inhibits quorum development in the host (Figure S1).

### 3.7. Effect of Cinnamaldehyde on Phagocytosis and Nitric Oxide Production in Rabbit Corneal Cell Lines (HCEC)

**Figure 6:**
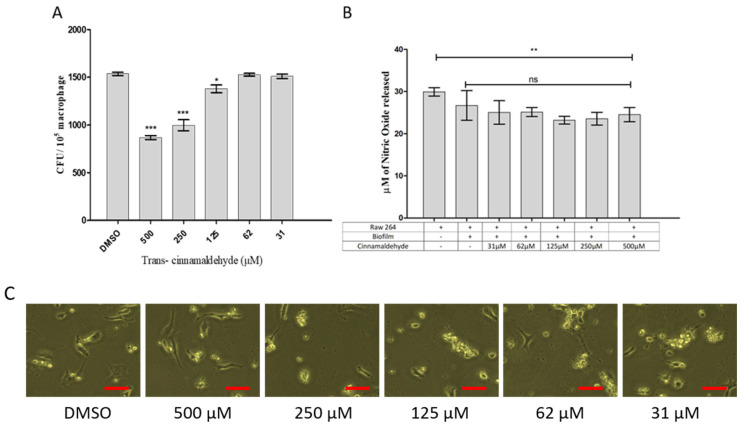
CA’s impact on the intracellular survival of bacteria that have been dislodged from *K. pneumoniae* biofilm in HCEC epithelium cell lines. (**A**). RAW 264.7 macrophage cell lines were co-cultured with biofilm *K. pneumoniae* after it was treated with CA 31 to 500 µM concentration. CFU per 10^5^ macrophages was used to express the quantity of live bacteria found 90 min after inoculation. The mean ± standard error of triplicate values is shown in the preceding data according to Student’s *t* test analysis. (**B**). The impact of cinnamaldehyde on the generation of nitric oxide in RAW264.7 macrophages co-cultured with *K. pneumoniae* biofilm. Cinnamaldehyde was used to cure the *K. pneumoniae* biofilm on Raw264.7 macrophages. The Griess reagent (1% sulfanilamide/0.1% naphtyl ethylene diamine dihydrochloride in 2.5% H_3_PO_4_) was used to measure the amount of NO in the culture supernatant following a 6 h incubation period. (**C**) The phase contrast image of RAW 264.7 cell lines with and without *K. pneumoniae* infection. The red line scale bar represents 50 µM. The mean ± standard error of triplicate values is shown in the data above. ns (Not significant) * *p* ≤ 0.05; ** *p* ≤ 0.01; *** *p* ≤ 0.001, difference from the control group as determined by Student’s *t* test.

Cinnamaldehyde, at concentrations ranging from 125 to 500 µM, was found to have the capacity to induce the clearance of phagocytosed bacterial cells of *K. pneumoniae* biofilms (*p* ≤ 0.05, Figure 5A). According to the findings, although CA treatment decreased NO generation as compared to untreated raw macrophages, it did not affect NO generation in macrophages when exposed to *K. pneumoniae* biofilms (Figure 6). This implies that cinnamaldehyde can modulate macrophages, indicating macrophage activation, the ensuing inflammation, and eventual pathogen clearance via an alternate mechanism.

### 3.8. Cytokine Responses of HCEC Corneal Epithelial Cell Lines to K. pneumoniae Biofilms and CA Treatment

**Figure 7:**
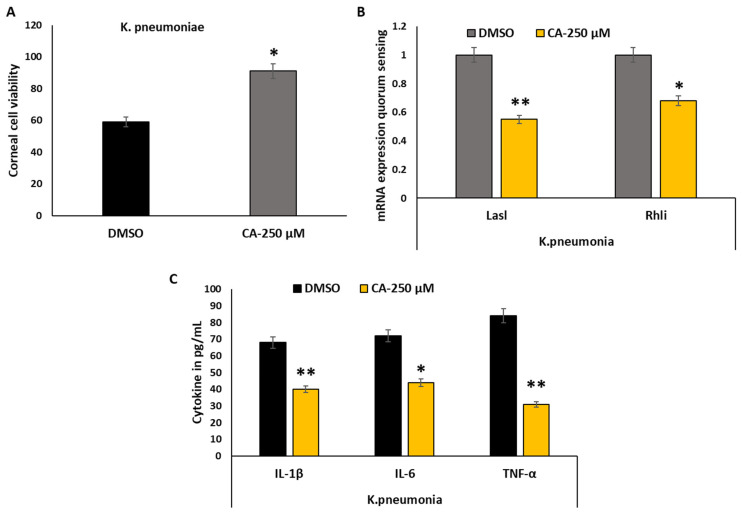
Effect of CA on corneal cell lines and it’s cytokine gene expression using qRT-PCR. (**A**) Corneal epithelial cell (HCEC) viability following exposure to *K. pneumoniae* in the presence of DMSO (vehicle control) or cinnamaldehyde (CA-250 µM). CA treatment significantly improved corneal cell viability compared with the control. (**B**) Relative mRNA expression of quorum-sensing regulatory genes (*LasI* and *RhlI*) in *K. pneumoniae* biofilms following treatment with CA-250 µM compared with DMSO control, as determined by qRT-PCR. (**C**) Pro-inflammatory cytokine production (IL-1β, IL-6, and TNF-α) in HCEC cells infected with *K. pneumoniae*, measured by ELISA following treatment with CA-250 µM or DMSO control. The results were considered significant for * *p* < 0.05; ** *p* < 0.01.

The release of cytokines by HCEC corneal epithelial in reaction to the microbe’s detection and phagocytic death is an additional feature of host-microbial interaction. Treatment with CA-250 μM treatment interact with live *K. pneumoniae* biofilms and examined the expression of mRNA that encodes pro- and anti-inflammatory cytokines to see if this held true for corneal cell interaction with *K. pneumoniae* biofilms as well. As can be observed from the results, the corneal cell lines significantly increased the cell viability during the CA treatment (Figure 7A) and there was a significant decrease in the expression of the pro-inflammatory cytokines (IL-β1, IL-6, and IFN-γ) when corneal cells were co-cultured with live biofilms of *K. pneumoniae*. Based on experimental data shown in the provided figure, cinnamaldehyde treatment (250 μM) consistently reduces mRNA expression of key QS genes across multiple pathogenic bacteria: *LasI* and *RhlI* genes show decreased expression in *K. pneumoniae*. The reduction appears particularly pronounced for *RhlI* expression across all three species. Cinnamaldehyde treatment significantly upregulated mRNA expression of pro-inflammatory cytokines, including IL-1β, IL-6, and IFN-γ (Figure 7C). Additionally, CA exhibited notable quorum-sensing (QS) inhibitory activity by suppressing key QS genes, such as *LasI* and *RhlA* (Figure 7B). In QS reporter strains, GFP production decreased by approximately 70% at the highest tested concentration of CA (250 µM). Macrophages co-cultured with *K. pneumoniae* biofilms demonstrated elevated TNF-α mRNA levels, whereas CA treatment mitigated this biofilm-induced TNF-α response in corneal cells (Figure 7C). Furthermore, mRNA levels of the anti-inflammatory cytokine IL-10 were increased in macrophages co-cultured with live biofilms of *S. aureus*, *P. aeruginosa*, and *K. pneumoniae*, suggesting a modulatory effect of biofilms on the host immune response.

Combination treatments reveal additive activity of CA-250 μM (75.2% biofilm inhibition). Treatment with cinnamaldehyde (250 μM) significantly reduces pro-inflammatory cytokine production, including IL-1β, IL-6, and TNF-α, across *K. pneumoniae* infections, while maintaining corneal cell viability above 80%. However, unlike the pro-inflammatory cytokines, the use of cinnamaldehyde treatment did not up-regulate the expression of inflammatory markers (Figure 7C).

## 4. Discussion

*Klebsiella* spp. are notorious pathogens equipped with virulence factors that enable their colonization and persistence within host tissues. These virulence factors, including capsular polysaccharides (CPS) and type 1 and 3 fimbriae, play a crucial role in biofilm formation on medical devices, leading to device-related infections [30]. This study investigated the potential of cinnamaldehyde to inhibit biofilm formation by *S. aureus*, *P. aeruginosa*, and *K. pneumoniae*. Additionally, the immune-modulating properties of cinnamaldehyde were evaluated during the biofilm stage using an in vitro macrophage model based on RAW 264.7 cells. The findings highlight the potential of cinnamaldehyde as a promising therapeutic agent for treating biofilm-related infections by targeting bacterial resistance and enhancing host immune responses. Biofilms are antibiotic- and disinfectant-resistant, making them a major healthcare concern [31]. Understanding biofilm formation mechanisms and assessing biofilm avoidance measures requires accurate biofilm measurement [32]. Cinnamaldehyde demonstrated a clear dose-dependent inhibition of *S. aureus*, *P. aeruginosa*, and *K. pneumoniae* biofilm formation, as evidenced by both crystal violet staining and XTT reduction assays. However, lower concentrations of cinnamaldehyde (below 125 µM) did not show any significant effect on the clearance of phagocytosed bacterial cells in the biofilm stage. These results also indicate that CAD in biofilm-Raw macrophage cells more effectively influenced the clearance of phagocytosed *S. aureus*, *P. aeruginosa*, and *K. pneumoniae* cells. This investigation highlights that cinnamaldehyde has the potential to enhance the clearance of phagocytosed bacterial cells in biofilm forms within macrophages. The dose-dependent effect observed also revealed that cinnamaldehyde may have a more pronounced impact on the clearance of phagocytosed bacteria. These findings contribute to our understanding of CAD immunomodulatory properties and its potential as a therapeutic agent in combating *S. aureus*, *P. aeruginosa*, and *K. pneumoniae* infections. Fluorescence microscopy further supported these findings by visualizing the decreased biofilm density and structure. The Acridine orange (AO) diffuses through intact cytoplasmic membranes in living cells, where it interacts with DNA, emitting bright green fluorescence. These examinations also differentiate between live and dead cells in the tested well plate. CA inhibited the formation of *S. aureus*, *P. aeruginosa*, and *K. pneumoniae* biofilms in a dose-dependent manner. Parallel results explored as cinnamaldehyde analog anti-biofilm effects against *Enterococcus faecalis* [17], *Haemophilus influenza* [33]; *S. mutans* [34] *L. monocytogenes* [35] *P. aeruginosa* [36], *K. pneumonia* [37].

The expression levels of the gene-encoding type 3 filaments and c-Di-GMP production are intimately linked to the biofilm development of *S. aureus*, *P. aeruginosa*, and *K. pneumoniae* [38]. CA significantly reduced the expression of genes involved biogenesis of type 3 fimbriae (*mrkH*, *mrkA*, *mrkB*, *mrkC*, *mrkF*), c-Di-GMP degradation gene (*mrkJ*), and regulatory mechanisms (bolA). It has been shown that BolA is necessary for *K. pneumonia* to produce siderophores, form biofilms, and adhere to surfaces [39,40]. In addition, Cinnamaldehyde significantly reduced the expression of genes involved in siderophore synthesis (*ybtS*). *S. aureus*, *P. aeruginosa*, and *K. pneumoniae* maintain intracellular bacterial persistence by controlling macrophage iron metabolism to get iron when confined within the host cell [41]. CA, by inhibiting siderophore production, may limit the availability of iron to *S. aureus*, *P. aeruginosa*, and *K. pneumoniae*, hindering its growth and biofilm formation. Type 3 fimbriae are filamentous structures that help bacteria stick to host cells and surfaces [42]. By interfering with fimbriae biogenesis, CA can prevent stable biofilm formation [43]. In contrast, CA showed no effect on the expression of the type 3 fmbriae gene (*mrkD*) and c-Di-GMP synthesis gene (*yfiN*). This clears that anti-biofilm property of cinnamaldehyde is mediated by the down steam targets of c-Di-GMP rather than its synthesis.

A rise in c-Di-GMP has the ability to attach to the biofilm switch protein MrkH, which has transcriptional regulators that bind to the mrkA promoter upstream and activate mrkABCDF [44]. Hence, we postulated that anti-biofilm property of CA might have been mediated by its competitive binding to MrkH protein. Computational docking is an influential method employed for predicting the binding interactions between molecules [45]. The results revealed that both cinnamaldehyde and c-Di-GMP may compete for the same binding site on the protein. Mutation of c-Di-GMP binding site amino acids in MrkH protein were reported with defective in binding to its target DNA [34]. Recent computational study using machine learning and molecular simulation reported antimicrobial peptides that bind to mrkH as novel antibiofilm candidates against potential inhibitor against *S. aureus*, *P. aeruginosa*, and *K. pneumoniae* biofilm.

The phagocytic clearance of microorganisms associated with *S. aureus*, *P. aeruginosa*, and *K. pneumoniae* biofilm by corneal epithelial cell line HCEC was enhanced by cinnamaldehyde. In response to ocular biofilms, CA markedly altered macrophage cytokine responses. In part through controlling phagocytic activity of macrophage and microbicidal activity, nitric oxide (NO) is essential for antibacterial host defense against *S. aureus*, *P. aeruginosa*, and *K. pneumoniae* [44]. On the other hand, the results from the present study revealed that both NO level and iNOS gene expression were decreased by the biofilm of *K. pneumonia*. Even the CA treatment failed to augment the NO level and iNOS gene expression in the macrophage that was co-cultured with *K. pneumonia*. Based on these results, CA may enhance the clearance of microorganisms associated with biofilms by modifying macrophage activity, possibly through pathways other than nitric oxide generation. Inhibition of NO production under *S. aureus*, *P. aeruginosa*, and *K. pneumoniae* infection increased the levels of inflammatory cytokine TNFα without affecting inflammatory cell profiles [46]. Similarly, it serves as a well-characterized model pathogen for biofilm formation and antibiotic resistance, which are major clinical concerns. It provides insights into the interactions between biofilm-producing bacteria and host immune responses, which are relevant to keratitis management. The experimental data can shed light on potential adjunctive therapies, such as cinnamaldehyde, targeting biofilms and immune modulation, applicable across multiple bacteria. The present study also revealed an increase in expression of TNFα in macrophages that were co-cultured with *K. pneumonia* biofilms. Although advantageous for the host in the early stages of an infection, inflammatory immune responses may prove to be harmful as the infection progresses. CA treatment counteracts the deleterious effects of biofilm-induced inflammation by significantly decreasing the expression of TNFα mRNA and by increasing the expression of mRNA-encoding an anti-inflammatory cytokine IL-10. On the other hand, the decline in the levels of mRNA-encoding pro-inflammatory cytokines (IL-1β, IL-6) caused by biofilm was reversed by cinnamaldehyde treatment. A recent study revealed documented down regulation of IL-1β and IL-6 at both mRNA and protein level is responsible for lower internalization rate of hyper virulent strain of *K. pneumoniae* in RAW 264.7 cells [47]. One of the most researched pro-inflammatory cytokines in the fight against *S. aureus*, *P. aeruginosa*, and *K. pneumoniae* infection is IFN-γ and biofilms are reported to decrease IFN-γ production [48]. A Streptomyces-derived bioactive compound, ASK2, was earlier reported to induce TNF-α and IFN-γ and possess antibiofilm property against *S. aureus*, *P. aeruginosa*, and *K. pneumoniae* infections [47]. Apart from IFN-γ, IL-6 also plays a significant role in promoting survival after sepsis and *K. pneumoniae* infection. It has been revealed that the activation of macrophages and other innate immune responses are mediated by IFN-γ/STAT/IRF-1 signaling in response to *S. aureus*, *P. aeruginosa*, *K. pneumoniae* and *K. pneumoniae* [39]. While this study demonstrates significant antibiofilm and immunomodulatory effects of cinnamaldehyde against bacterial keratitis pathogens, certain limitations should be acknowledged. Primarily, the experimental work was conducted under in vitro conditions using cell culture and biofilm models, which do not fully replicate the complex in vivo ocular environment including immune system factors and drug bioavailability [48]. Additionally, the lack of in vivo validation in animal models limits the direct translational application of our findings at this stage. Future studies should focus on detailed pharmacokinetic and toxicity profiling in appropriate animal models, as well as investigate the therapeutic efficacy of optimized cinnamaldehyde-based formulations in vivo. Furthermore, coupling ligand binding assays with the molecular docking results would strengthen mechanistic insights into cinnamaldehyde’s molecular interactions. Exploring synergistic effects with existing antimicrobials could also further enhance clinical relevance and therapeutic performance [48,49]. Based on these results, cinnamaldehyde may help remove biofilms by increasing macrophage activation and cytokine production.

## 5. Conclusions

This study demonstrates the dual functionality of cinnamaldehyde as both an anti-biofilm and attenuates pro-inflammatory cytokine responses in association with reduced adhesion and biofilm formation agent. It downregulates key genes in the c-di-GMP–type 3 fimbriae axis and enhances macrophage-mediated clearance of *K. pneumoniae* in vitro. Furthermore, CA modulates the macrophage cytokine profile, promoting a pro-inflammatory response while mitigating excessive inflammation. These findings suggest that CA is a promising candidate for treating biofilm-associated *S. aureus*, *P. aeruginosa*, and *K. pneumoniae* infections, though further mechanistic studies are required to fully elucidate its immunomodulatory pathways.

## Figures and Tables

**Table 1:** List of biofilm formation gene primer sequences used for qRT-PCR.

Gene	Forward Primer	Reverse Primer	Amplicon Size (bp)
*yfiN*	TACGTACCGCGCTACATGAC	TCGGGCATCGGAATTGTTCA	95
*mrkA*	GCAAACTGGGCGTAAACTGG	CTTTCGCTTTCGGCTGAGTG	186
*mrkB*	ACCCGCTTTATTTATCCAGG	AAACGGGGTGGTAATGGTAT	138
*mrkC*	GGTATCAACGGTTCGCTGGA	CCAATGCCGCTCTGACGATA	170
*mrkD*	AACGTGCCGGGAATTGGTAT	GTGGTTGCCGCAGTTTTGAT	155
*mrkF*	AACGAAAACGCCGGGTATCT	CCTGCAAACGCACCTGATTT	148
*mrkJ*	CGAGCCACAGTGAGGTATCC	CCTGCGTCCATTTCGAGGTA	96
*mrkH*	TGGACTTTGCCGAGTT	ACCGCTATTGTCATGTTT	120
*BolA*	GCCATTGAGTTTGCGGTCTC	GTCGGGTGAAAAAGCAGCAG	128
*ybtS*	GACGGAAACAGCACGGTAAA	GAGCATAATAAGGCGAAAGA	242
16S rRNA	GATGACCAGCCACACTGGAA	CTGCAGGGTAACGTCAATCG	194

*yfiN*—diguanylate cyclase; *mrkA*—Type 3 fimbrial shaft; *mrkB*—chaperone; *mrkC*—outer membrane usher; *mrkD*—fimbrial adhesion; *mrkF*—assembly of the filament; *mrkJ*—Phosphodiesterase; *mrkH*—c-di-GMP-dependent transcriptional activator; *BolA*—transcriptional regulator; *ybtS*—Siderophore.

**Table 2:** List of immune response gene primer sequences used for qRT-PCR.

Gene	Forward Primer	Reverse Primer	Amplicon Size (bp)
*LasL*	5′-CGTGCTCAAGTGTTCAAGG-3′	5′-TACAGTCGGAAAAGCCCAG-3′	170
*RHIi*	5′-TTCATCCTCCTTTAGTCTTCCC-3′	5′-TTCCAGCGATTCAGAGAGC-3′	177
*GAPDH*	CGCTAACATCAAATGGGGTG	TTGCTGACAATCTTGAGGGAG	201

## Data Availability

The original contributions presented in this study are included in the article/Supplementary Materials. Further inquiries can be directed to the corresponding authors.

## Supplementary Materials

The following supporting information can be downloaded at https://www.mdpi.com/article/10.3390/pharmaceutics18010005/s1: Figure S1: Antimicrobial activity of CA on *K. pneumoniae* and *S. aureus*.
