# Supplementary material for: Antibiofilm and Immunomodulatory Effects of Cinnamaldehyde in Corneal Epithelial Infection Models: Ocular Treatments Approach
Source: Pharmaceutics. 2025 Dec 19;18(1):5. doi: 10.3390/pharmaceutics18010005 (PMC12845009; doi:10.3390/pharmaceutics18010005)
Supplement: Supplementary file 1 [file pharmaceutics-18-00005-s001.zip › pharmaceutics-3993115-supplementary.pdf]

pharmaceutics

Supplementary Materials: Antibiofilm and Immunomodulatory Effects of Cinnamaldehyde in Corneal Epithelial Infection Models: Ocular Treatments Approach

Ashraf Khalifa, Muthukumar Thangavelu, Hairul-Islam M Ibrahim and Krishnaraj Thirugnanasambantham

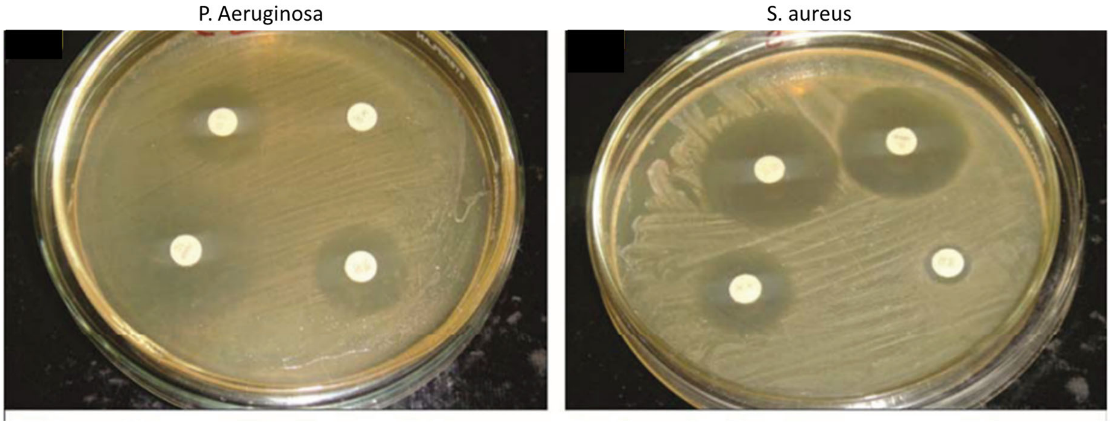

Figure S1. Antimicrobial activity of CA on K. pneumoniae and S. aureus.

 https://doi.org/10.3390/pharmaceutics18010005
